# HDAC inhibitors, MS-275 and salermide, potentiates the anticancer effect of EF24 in human pancreatic cancer cells

**DOI:** 10.17179/excli2016-186

**Published:** 2016-04-04

**Authors:** Atiye Seda Yar Saglam, Akin Yilmaz, Hacer Ilke Onen, Ebru Alp, Handan Kayhan, Abdullah Ekmekci

**Affiliations:** 1 Department of Medical Biology and Genetics, Faculty of Medicine, Gazi University, Besevler, Ankara, Turkey; 2 Department of Medical Biology, Faculty of Medicine, Hitit University, Çorum, Turkey; 3 Department of Medical Biology, Faculty of Medicine, Giresun University, Giresun, Turkey; 4 Department of Adult Heamatology, Faculty of Medicine, Gazi University, Besevler, Ankara, Turkey

**Keywords:** BxPC-3 cells, EF24, HDACI, MS-275, salermide, pancreatic cancer

## Abstract

Histone deacetylases (HDACs) play a major role in the regulation of chromatin structure and gene expression by changing acetylation status of histone and non-histone proteins. MS-275 (entinostat, MS) is a well-known benzamide-based HDACI and Salermide (SAL), a reverse amide compound HDACI, have antiproliferative effects on several human cancer cells. In this study, we aimed to investigate the effects of HDACIs (MS and SAL) alone and/or combined use with EF24 (EF), a novel synthetic curcumin analog, on human pancreatic cancer cell line (BxPC-3). *In vitro, *BxPC-3 cells were exposed to varying concentrations of MS, SAL with or without EF, and their effects on cell viability, acetylated Histone H3 and H4 levels, cytotoxicity, and cleaved caspase 3 levels, and cell cycle distribution were measured. The viability of BxPC-3 cells decreased significantly after treatment with EF, MS and SAL treatments. MS and SAL treatment increased the acetylation of histone H3 and H4 in a dose dependent manner. MS and SAL alone or combined with EF were increased the number of cells in G1 phase. In addition, treatment with agents significantly decreased the ratio of cell in G2/M phase. There were significant dose-dependent increases at cleaved Caspase 3 levels after MS treatment but not after SAL treatment. Our results showed that HDAC inhibitors (MS and SAL), when combined with EF, may effectively reduce pancreatic cancer cell (BxPC-3) progression and stop the cell cycle at G1 phase. Further molecular analyses are needed to understand the fundamental molecular consequences of HDAC inhibition in pancreas cancer cells.

## Introduction

Pancreas cancer is one of the most lethal malignancy seen in humans and fourth among death due to cancer in both sexes (Siegel et al., 2016[34]). Molecular basis of pancreatic cancer development and progression includes both genetic and epigenetics alterations (Lomberk and Urrutia, 2015[21]). Gene expression is controlled by epigenetic mechanisms such as DNA methylation, histone modifications and non-coding RNAs (Holoch and Moazed, 2015[11]) Histone modifications alter chromatin structure by affecting the functional interactions between the histones or the connection of histones with DNA (Kouzarides, 2007[15]). Acetylation of histones is associated with a transcriptionally active chromatin structure among all known modification. The acetylation status of histones is regulated by two enzyme families; histone acetyltransferases (HATs) and histone deacetylases (HDACs) (Di Gennaro et al., 2004[5]). The HDAC family consists of eighteen different human HDAC isoforms that are grouped into four classes; class I (HDAC1-3 and 8), class IIa (HDAC4, 5, 7 and 9), class IIb (HDAC6 and 10), class III [sirtuins, (SIRT1-7)] and class IV (HDAC11) (Di Gennaro et al., 2004[5]; Kouzarides, 2007[15]).

HDAC inhibitors (HDACIs) are classified according to their chemical structures and there are five classes of them including short-chain fatty acids, hydroxamic acids, cyclic tetrapeptides, aliphatic acids and benzamides, MS-275 (MS), also called entinostat, is a benzamide type HDAC inhibitor (HDACi) and specific for class I HDACs (Khan and La Thangue, 2012[13]). Its antiproliferative effects has been shown in several cancer cell lines including cholangiocarcinoma (Baradari et al., 2007[2]), leukemia (Rosato et al., 2003[31]; Gao et al., 2008[10]), pediatric solid tumors (Jaboin et al., 2002[12]), glioblastoma (Eyüpoglu et al., 2006[7]), prostate cancer (Khandelwal et al., 2008[14]; Qian et al., 2007[28]), hepatoma (Gahr et al., 2008[9]), bladder (Qu et al., 2010[30]), and pancreas (Peulen et al., 2013[26]).

Sirtuins belongs to the Class III HDACs. SIRT1 and SIRT2 have roles in several cellular processes such as metabolic regulation, genomic stability, DNA repair, epigenetic silencing and chromatin modification (Finkel and Deng, 2009[8]). Their main substrates are histone proteins and dysregulation of SIRT1 and SIRT2 expression is frequently found in several cancers (Roth and Chen, 2014[32]). Salermide (SAL), an inhibitor of SIRT1 and SIRT2, is able to kill tumor cells (Lara et al., 2009[17]) including leukeamia, lymphoma, colon, breast (Lara et al., 2009[17]), lung (Liu et al., 2012[19]), neuroblastoma and pancreatic cancer cells (Liu et al., 2013[20]). After exposure of cancer cells to SAL, epigenetically silenced proapoptotic genes are reactivated and thus, it generates antiproliferative effects on cancer cells (Lara et al., 2009[17]).

EF24 (EF), a synthetic curcumin analog (Adams et al., 2004[1]), alone or combined with other agents shows anticancer effects on several tumor cell lines including mesothelioma (Onen et al., 2015[23]), ovarian (Tan et al., 2010[37]), colon (Subramaniam et al., 2008[35]), prostate (Yang et al., 2013[39]), and breast (Sun et al., 2009[36]). With regard to the pancreatic cancer cells (MiaPaCa-2 and PANC-1), Lagisetty et al. (2012[16]) showed the antiproliferative effect of EF. Like curcumin, EF targets Nuclear Factor Kappa B (NF-κB) signaling pathway and induce programmed cell death (Yang et al., 2013[39]).

The presence of HDACs (HDAC1-3) expression in human pancreatic cancer specimens was shown by immunohistochemical analysis (Nakagawa et al., 2007[22]). Moreover, aberrant expression of class 1 HDACs (HDAC1, HDAC3) and class 2 (HDAC7) was demonstrated in BxPC-3 cells (Ouaïssi et al., 2008[24], 2012[25]).

Lehmann et al. displayed notably association between increased class I HDAC expression and elevated nuclear translocation of RelA/p65 (Lehmann et al., 2009[18]). Moreover, Weichert et al. (2007[38]) showed a significant correlation between up-regulated RelA/p65 expression and induction of the NF-κB pathway in pancreatic cancer patients. It is also known that SIRT1 physically associates with the NF-κB complex (mainly RelA/p65 subunit), and suppress mRNA synthesis via deacetylating RelA/p65 (Yeung et al., 2004[40]).

To our knowledge, the impact of EF alone or in combination with HDACIs (MS and SAL) on pancreatic cancer cell line (BxPC-3) has not been analyzed so far and thus we aimed to study possible effects of these agents by virtue of testing cell viability, histone acetylation, cell cycle analysis, caspase 3 activation and lactate dehydrogenase release. 

## Materials and Methods

### Chemicals

The reagents and kits used in this study were purchased from the following suppliers: SAL (N-{3-[2-hydroxynaphthalen-1- ylmethylene)-amino]-phenyl}-2-phenylpropionamidea, MS, EF, 3-(4,5-dimethylthiazol-2-yl)-2,5-diphenyl tetrasodium bromide (MTT), dimethyl sulfoxide (DMSO) and CelLytic from Sigma-Aldrich (St. Louis, MO, USA); Roswell Park Memorial Institute (RPMI)-1640 medium, fetal bovine serum (FBS), penicillin/streptomycin from Gibco (Grand Island, NY, USA); all of other chemicals and reagents from Sigma-Aldrich (St. Louis, MO, USA).

### Cell lines and culture conditions

The human pancreatic cell line, BxPC-3 (CRL-1687), was obtained from the American Type Culture Collection (ATCC, Manassas, VA, USA) and grown in RPMI-1640 medium supplemented with 10 % FBS and 1 % penicillin/streptomycin, at 37 °C in a humidified atmosphere containing 5 % CO_2_. When BxPC-3 cells were 80 % confluent, they were sub-cultured to fresh medium. The cultures were incubated for 24 h before the experimental treatments. 

SAL, MS and EF were dissolved in 100 % DMSO to make 1 mM stock solutions, kept at -20 °C, and then diluted in the cell culture medium immediately prior to use. In all experiments, control cells were incubated in media supplemented with DMSO alone. The final concentration of DMSO was maintained at 0.2 % and this concentration has no effect on growth and survival of BxPC-3 cells.

### Viability assay

Cell viability was determined after alone and combinational treatment with SAL and/or MS at various concentrations with or without selected dose of EF (1 µM) by MTT assay. BxPC-3 cells were seeded at density of 5 × 10^3^ cells/well in 96-well plates and incubated overnight at 37 °C. This cell line was incubated with various concentrations of SAL (2.5 to 120 µM) and MS (2.5 to 75 µM) with selected dose of EF for 24 h. After incubation with these agents, 10 μl of 5 mg/ml MTT in phosphate-buffered saline (PBS) was added to each well and the plates were incubated for an additional 4 h in the dark at 37 °C. The supernatants were aspirated, and formazan crystals were solubilized in 100 μl DMSO at 37 °C for 10 min with agitation. The absorbance of each well was measured at 570 nm using Spectramax M3 microplate reader (Molecular Devices, Silicon Valley, California, USA). All experiments were performed in quadruplicate for each dose.

### Acetylated Histone H3 and Acetyl-Histone H4 Sandwich ELISA assay

Enzyme-linked immunosorbent assay (ELISA) was used to detect specifically endogenous levels of acetylated lysines on histones H3 and H4 in BxPC-3 cells in presence of MS and SAL at 24 h. PathScan^® ^Acetylated Histone H3 and Acetyl-Histone H4 Sandwich ELISA Kits from Cell Signaling Technology were used according to the manufacturers' instructions. Absorbance of each well was measured using Spectramax M3 microplate reader (Molecular Devices) at 450 nm. The absorbance of each sample was divided by the absorbance of the untreated cell with the same incubation time to calculate a control index. All experiments were carried out in triplicate.

### Flow cytometric analysis of the cell cycle

BxPC-3 cells (1 × 10^6^) were treated with SAL and/or MS at various concentrations with or without 1 µM EF for 24 h. After trypsinization, detached cells were washed with PBS and then processed with a CycleTest-Plus^TM^ DNA reagent KIT (Becton Dickinson and Company BD Biosciences, San Jose, CA, USA). DNA QC particles (Becton Dickinson) were used as controls. The samples were analyzed using FACScan (Becton Dickinson) and the data were collected by Cell Quest (Becton Dickinson) and analyzed by use of FCS Express 5 Flow Research Edition software. These experiments were carried out in triplicate and independently repeated at least three times.

### Cytotoxicity assay

The lactate dehydrogenase (LDH) Cytotoxicity Detection Kit Plus (Roche Applied Science, Mannheim, Germany) was used to measure the cytotoxicity in MS, SAL and/or EF-treated BxPC-3 cells. Briefly, cells were treated with selected concentrations of MS and SAL with or without 1 µM EF for 24 h. After treatment of BxPC-3 cells with agents at various concentrations, this assay was performed according to the manufacturer's instructions. Optical density (OD) was measured following incubation at 490 nm using Spectramax M3 microplate reader (Molecular Devices, USA). 

### Detection of cleaved caspase 3 level

The presence of cleaved caspase-3 protein levels in BxPC-3 cells, which are important signs of apoptosis, was detected using the PathScan^®^ Cleaved Caspase-3 (Asp175) Sandwich Elisa Kit (Cell Signaling Technology Inc., Beverly, MA, USA). Briefly, after being treated with agents at indicated concentrations for 24 h, cell lysates were obtained and then protein content of the cell lysates was determined using the bicinchoninic acid (BCA)™ protein assay kit (Pierce, Rockford, Illinois, USA). This assay was performed according to the the manufacturer's protocols. At the end of this period, the absorbance of the cleaved Caspase-3 levels was measured at 450 nm with Spectramax M3 microplate reader (Molecular Devices, USA).

### Statistical analysis

All the quantitative data were presented as the mean values ± standard deviations (SD). Statistical comparisons among groups were performed by Student's **t-**test or one-way analysis of variance (ANOVA) followed by the Tukey test for multiple comparison. The values of p < 0.05 were considered as significant (marked with asterisks in the figures).

## Results

### Effects of MS, SAL and EF on the BxPC-3 cell viability

The BxPC-3 cells were treated with increasing concentrations of EF, MS and SAL and the effects on cell viability were assessed by enzymatic reduction of MTT. BxPC-3 cell lines were administered EF (0.5-60 μM), MS (2.5-25 μM), and SAL (5-100 μM), and dose-dependent effects of these agents on the cells were defined after 24 h. 

**Figure 1:**
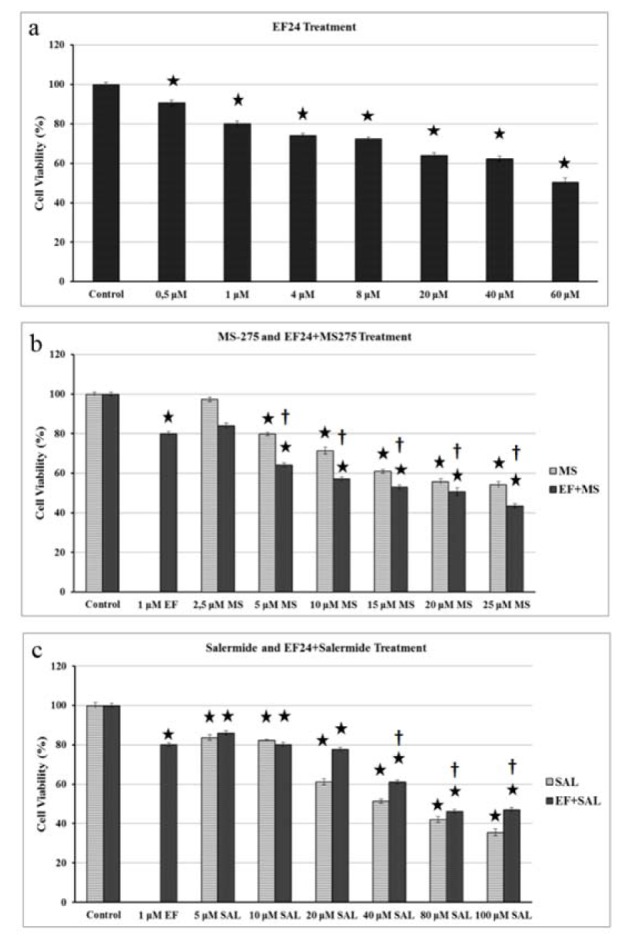
Effect of MS and SAL alone or in combination with EF on the viability of pancreatic cancer cells. Statistical significance was determined using two-way analysis of variance, a: EF alone, b: MS alone and MS+EF treatment, c: SAL alone and SAL+EF treatment. MS: MS-275; SAL: Salermide; EF: EF24. * p < 0.001, versus control cells for 24 h † p < 0.001, versus EF treatment for 24 h

As shown in Figure 1a(Fig. 1), the viability of BxPC-3 cells decreased significantly after treatment with EF. Moreover MS and SAL alone or with EF treatment was also shown to decrease the cell viability in a concentration-dependent manner (Figure 1b and 1c(Fig. 1), respectively). The half-maximal inhibitory concentration (IC_50_) values of MS was about 25 µM and SAL was 40 µM for 24 h. IC_50_ value for EF was found to be 60 µM. 

Then cells were treated with 1 µM EF plus different doses of MS or SAL and cell viability was assessed after 24 h by MTT. Combined treatment of 1 µM EF with MS enhanced the antiproliferative effect of MS at all concentrations and it was clearly reduced the IC_50_ concentration of MS up to 10 µM (Figure 1b(Fig. 1)).

However, combined treatment of 1 µM EF with SAL attenuated the inhibitory effect of SAL alone treatment and increased the IC_50_ concentration of SAL about 80 µM (Figure 1c(Fig. 1)).

### Determination of Acetylated Histone H3 and Histone H4 Levels

**Figure 2:**
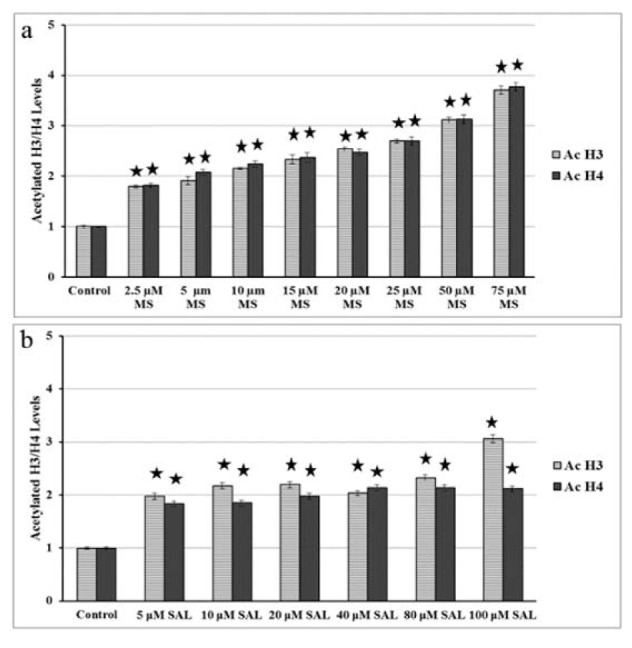
Acetylated histone H3 and H4 levels in BxPC-3 cells treated with MS (a) and SAL (b) alone or in combination with EF for 24 h. MS: MS-275; SAL: Salermide; EF: EF24 *p < 0.001.

Treatment of BxPC-3 cells with different concentration of MS (2.5-75 µM) (Figure 2a(Fig. 2)) and SAL (5-100 µM) (Figure 2b(Fig. 2)) alone was found to increase both acetylated histone H3 and H4 levels. MS increased the acetylation of histone H3 and H4 in a dose dependent manner. At the highest MS concentration (75 µM), 3.5 fold increased acetyl histone H3 and H4 was found after 24 h.

For the SAL treatment, dose dependent increase in the acetylation status of histones was not found like in MS treatment. Most of the SAL doses mainly elevated the histone H3 and H4 acetylation up to 2 fold (Figure 2b(Fig. 2)).

### Cytotoxic effect of MS and/or SAL alone and combination treatment with EF

**Figure 3:**
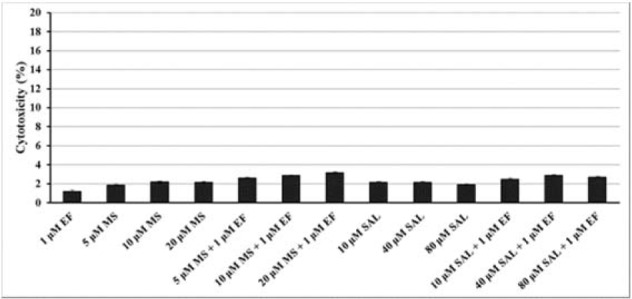
Cytotoxicity of MS and SAL alone or in combination with EF evaluated by LDH release from BxPC-3 cells after 24 h of incubation. LDH: lactate dehydrogenase; MS: MS-275; SAL: Salermide; EF: EF24

The effects of three concentrations of MS (5, 10 and 20 µM) and SAL (10, 40 and 80 µM), as single agents and combined treatment with 1 µM EF, were evaluated on BxPC-3 cells for 24 h using LDH release to culture medium (Figure 3(Fig. 3)). For all concentrations of the agents cytotoxicity ratio were not exceed 3.5 %.

### Cell cycle perturbation by HDACI and their combinations with EF

**Figure 4:**
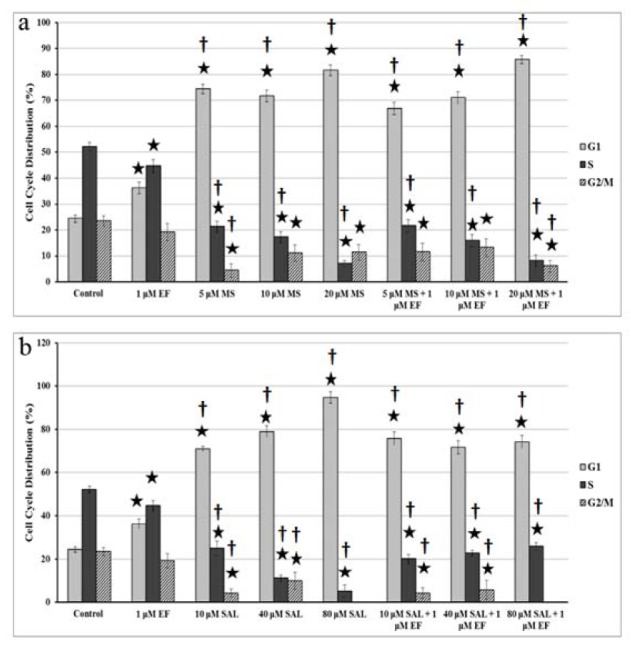
Effect of MS (a) and SAL (b) alone or in combination with EF on cell cycle by PI incorporation. Percentage of cells in each phase of the cell cycle (G1, S, and G2-M) is indicated. MS: MS-275; SAL: Salermide; EF: EF24. * p < 0.001, versus control cells for 24 h † p < 0.001, versus EF treatment for 24 h

MS, SAL and EF significantly affected to BxPC3 cell cycle progression as compared to the control cells at 24 h (Figure 4(Fig. 4)). In flow cytometry analysis, the percentage of control BxPC-3 cells in each phase was stable. In addition, MS and SAL produced a consistent increase of cells in the G1 phase of cell cycle in BxPC-3 cell lines. Moreover, combined treatments of MS and SAL with EF were also increased the number of cells in G1 phase. As seen in Figure 4(Fig. 4), treatment with agents also significantly decreased the ratio of cell in G2/M phase.

### Apoptosis induction by HDACI and their combinations with EF

**Figure 5:**
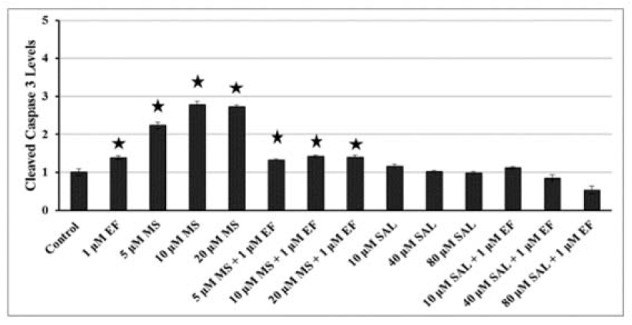
Effects of MS and SAL alone or in combination with EF on levels of caspase-3 cleavage in BxPC-3 cells, as measured by colorimetric assay at 24h. *p < 0.001, compared with control cells for 24 h. MS: MS-275; SAL: Salermide; EF: EF24

To investigate further whether the induction of apoptosis by MS and SAL is mediated by classical apoptotic pathways, we analyzed the levels of cleaved caspase 3, a well-known mediator of apoptotic pathways. There were significant dose-dependent increases (ranges from 2-fold to 3-fold) at cleaved Caspase 3 levels after MS for 24 h (p < 0.05) (Figure 5(Fig. 5)). When correspond to control cells, MS + EF administration increased the level of cleaved caspase 3 in BxPC-3 cells (p < 0.05, Figure 5(Fig. 5)). 

Cleaved caspase-3 protein levels decreased dose dependent manner with SAL in BxPC-3 cells (Figure 5(Fig. 5)). Moreover, the combination of SAL and EF caused decrease in cleaved caspase-3 levels in these cells after 24h, as compared to control cells (p > 0.05) (Figure 5(Fig. 5)). 

## Discussion

Expression profiles of the HDAC and SIRT enzyme types in pancreatic cancer cells and impact of HDACIs on their expression were analyzed in some studies. With this regard, mRNA expression level of HDAC (1-7) and class III (SIRT1-6) was shown in pancreatic cell lines (MiaPaCa-2, BxPC-3, PANC-1, SOJ-6). Compared with the mRNA expression level of HDAC (1-7), it was observed that diminished SIRT (1-6) mRNA expression level in BxPC-3 cells (Ouaïssi et al., 2008[24]). However, there was no difference between protein expression level of HDAC (1, 4, 7) and SIRT (1, 2) in BxPC-3 cells (Ouaïssi et al., 2008[24]). Moreover, Ouaïssi et al. (2012[25]) analyzed the effects of HDACi [TSA and panobinostat (LBH589)] on the mRNA expression of HDAC genes (HDAC 1-4, 7 and SIRT 1, 2) from the pancreatic cell lines (BxPC-3, PANC-1 and SOJ-6) and non-malignant pancreatic epithelial cell line (HPDE/E6E7). Both TSA and panobinostat has no impact on mRNA expression of HDAC genes in BxPC-3 cells. However, protein expression of four studied genes was either increased or decreased by LBH589 and TSA administration on BxPC-3 cells (Ouaïssi et al., 2012[25]). Due to the fact that apoptosis level was higher in TSA-treated BxPC-3 cells than those of cells treated with 5-aza-dC, histone deacetylation is suggested to take a part in the progression in BxPC-3 (Cai et al., 2011[3]). 

After inhibition of class I HDACs, it is suggested that genes controlled by NF-κB pathway can be activated to allow cancer cell proliferation. From this point, Peulen et al. (2013[26]) displayed that suppression of HDAC1 and HDAC3 promoted upregulation of COX-2 expression through activation of NF-κB pathway in BxPC-3 cells. So, inhibition of the NF-κB pathway using 1 µM EF decreased the cancer cells proliferation less than 60 % in pancreatic cancer cells (MiaPaCa-2 and PANC-1) (Lagisetty et al., 2012[16]). Similarly, our EF treatment of another pancreas cancer cell line (BxPC-3) also reduced the cell viability by 80 % (Figure 1a(Fig. 1)). Because of the relationship between class I HDACs and NF-κB pathway, we want to analyze the effect of dual inhibition of class I HDAC by MS and NF-κB pathway via EF on BxPC-3 cells. To our knowledge, such a dual inhibition targeting both class I HDACs and NF-κB pathway has not been investigated so far. 

*In vitro* analysis performed on pancreatic cancer cell lines showed different effects of HDACIs alone (Peulen et al., 2013[26]; Sato et al., 2004[33]) or in combination with classical chemotherapeutics (Qiao et al., 2013[29]) and these differences can be attributed to the characteristics of the cell line and/or the chemical properties of HDACi. HDACIs affect cell proliferation, cell cycle progression (Ouaïssi et al., 2008[24]; Qiao et al., 2013[29]; Chun et al., 2009[4]; Zhang et al., 2008[41]), gene expression (Chun et al., 2009[4]; Emonds et al., 2010[6]; Sato et al., 2004[33]) and also miRNA expression (Zhang et al., 2008[41]) in pancreas cancer cells.

Most of the analyzed HDACIs exert antiproliferative and apoptotic effects on pancreas cancer cells. Sato et al. (Sato et al., 2004[33]) displayed antiproliferative effect of HDACi (FR901228) in five human pancreatic cell lines (MiaPaCa-2, Capan-1, BxPC-3, HPAF and Panc-1). In addition, treatment of BxPC-3 cells with TSA caused stimulation of apoptosis by mitochondria-dependent pathway (Ouaïssi et al., 2008[24]). Moreover, it was shown that TSA treatment diminished the cell viability of BxPC­3 at various concentration (range 0.1-0.2 µmol/L) for 24 - 72 h. Compared with the control cells, elevated levels of apoptosis was also determined in TSA-treated BxPC-3 cells (Zhang et al., 2008[41]). Ouaïssi et al. (2012[25]) showed that following incubation with the TSA or LBH589, apoptosis induced in BxPC-3 cells in dose-and time-dependent manner, as well. Furthermore, LBH589 was shown to induce tumor regression in BxPC-3 xenografts (Ouaïssi et al., 2008[24]). Peulen et al. (2013[26]) showed that cell growth was inhibited in response to MS treatment on different human pancreas cancer cells (BxPC-3, CFPAC-1 and PANC-1) in a dose and time dependent manner. At 48 h, 1 μM concentration of MS diminished BxPC-3 cell viability by 50 % and 5 μM concentration of MS suppressed totally cell viability. Similar to above data, we found antiproliferative effects of MS on BxPC-3 cell line in a dose dependent manner (Figure 1b(Fig. 1)) 

With respect to the apoptotic effects of HDACIs, several studies showed molecular basis of programmed cell death seen after HDACi treatment. Following treatment of human pancretatic cancer cells (BxPC-3, CAPAN-1 and AsPC-1) with TSA resulted in upregulation of acetyl H3, p21Waf1 and Bax levels, increased phosphorylation of p38 and diminished phosphorylation of ERK 1/2 and AKT (Zhang et al., 2008[41]). In PANC-1 and BxPC-3 cells, it was also shown that apoptosis was correlated with the enhanced nuclear localization of survivin, up-regulated expression of BAX, and stimulation of caspase 3/7 (Chun et al., 2009[4]). Moreover, In five human pancreas cell lines (MiaPaCa-2, Capan-1, BxPC-3, HPAF and PANC-1), FR901228 triggered apoptosis through induction of caspase-3, degradation of survivin and p21^Waf-1 ^(Sato et al., 2004[33]). From these results, it is obvious that induction of caspase 3 is one of the common consequence seen after HDAC inhibitor exposure. Similarly, we found that MS treatment of BxPC-3 cells elevated histone H3 and H4 acetylation and cleaved caspase 3 levels (Figure 2a(Fig. 2) and 5(Fig. 5), respectively). But, Peulen et al. (2013[26]) was unable to show apoptosis in BxPC-3 cells after 24, 48 or 72 h of treatment with MS at doses ranges between 0.1-5 µM. 

HDACIs are capable of arresting cell cycle progression at different phases depending on the inhibitor and cell type. For example, Chun et al. (2009[4]) displayed that overexpression mRNA levels of p21^CDKN1A^ and p27^CDKN1B^ and diminished mRNA expression of cyclin D1 (CCND1) resulted in G_0_/G_1 _cell cycle arrest. In addition, after administration of TSA in BxPC-3 cells, decreased cell proliferation and G1 arrest were detected (Ouaïssi et al., 2008[24]; Zhang et al., 2008[41]). Moreover, it was shown that 50 % of the cells were arrested at the G1 phase by adding MS for 48 h (Peulen et al., 2013[26]). In concert with these findings, we also showed an increase in the number of G1 subpopulation of cells after MS treatment of BxPC-3 cells (Figure 4a(Fig. 4)). On the other hand, Sato et al. (2004[33]) displayed G2/M arrest in BxPC-3 cells after 24 h incubation of FR901228. Chidamide (CS0055/HBI-8000), a newly discovered HDACi, belongs to benzamide class was treated on pancreatic cancer cells (BxPC-1 and PANC-1) (Qiao et al., 2013[29]). After administration of this inhibitor on these cell lines, it was shown that cells arrested at G2/M phase of the cell cycle due to activation of p21 (Qiao et al., 2013[29]). 

While promising anticancer effects against pancreas cancer cell lines were shown for HDACIs, little is known how they affect patients. In a Phase I study, combination of entinostat, MS, with 13-cis retinoic acid (CRA) in patient with unresectable pancreatic cancer resulted stable disease up to six months. But, an objective response was not reported after this regimen (Pili et al., 2012[27]). Further phase studies are needed to clarify the beneficial impacts of HDACIs alone or combined regimens on cancer patients.

Lara et al. (2009[17]) analyzed the effects of SAL on normal fibroblast cells and several cancer cell lines including leukeamia, lymphoma, colon and breast. They showed a cell number reduction in all studied cancer cells, but not normal fibroblast cells. In these cells, SAL was also found to induce apoptosis with varying degrees in a cell-type and dose dependent manner. In another study, SAL was found to cause apoptotic cell death via endoplasmic reticulum stress induction in a dose and time dependent manner in human lung cancer cell lines (Liu et al., 2012[19]). In neuroblastoma (BE(2)-C) and pancreatic cancer cells (MiaPaca-2), the SIRT2 inhibitor SAL was shown to diminish the N-Myc and c-Myc protein levels, but not mRNA expression (Liu et al., 2013[20]). SAL reduced the pancreatic cancer cell (MiaPaca-2) number by 80 % at 25 µM concentration after 72h treatment (Liu et al., 2013[20]). Our results also showed that SAL reduced cell viability and caused cell cycle arrest at G1 phase in BxPC-3 cells, but not increased the cleaved caspases 3 levels (Figure 1c(Fig. 1), 4b(Fig. 4), and 5(Fig. 5), respectively).

Briefly, our results showed that HDACIs (MS and SAL), when combined with EF, may effectively reduce pancreatic cancer cell (BxPC-3) progression and stop the cell cycle at G1 phase. Further studies are needed to clarify the underlying molecular mechanisms of HDAC inhibition in pancreas cancer cells. This may be resulted in a development of more effective agents and promising therapy strategies for the patients with pancreas cancer.

## Acknowledgements

This study is partially supported by Gazi University Research Foundation (Project code 01/2010-39).

We would like to appreciate MSc. Zubeyir Elmazoglu for his sincere help. Without his contributions this study could not be performed.

## Conflict of interest

The authors declare that they have no conflict of interest.
